# The utility of human fallopian tube mucosa as a novel source of multipotent stem cells for the treatment of autologous reproductive tract injury

**DOI:** 10.1186/s13287-015-0094-1

**Published:** 2015-05-21

**Authors:** Jiaojiao Wang, Yong Zhao, Xiaoyun Wu, Shande Yin, Yunhai Chuai, Aiming Wang

**Affiliations:** Department for Gynaecology and Obstetrics, Navy General Hospital, PLA, Fuchengmen Road, No.6, Beijing, 100048 China; Jing-Meng Stem Cell Technology CO., Ltd, Shangdi East Road,No.5-2, Beijing, 100048 China

## Abstract

**Introduction:**

Fallopian tube, which is normally discarded in surgical procedures, has proven to be a source of mesenchymal stem cells (MSCs) with increasing evidence. However, fallopian tube mucosa, which can be acquired via non-invasive procedures, is a previously unknown source of MSCs. In the present study, we explored the existence of MSCs in the human fallopian tube mucosa and also compared multipotent stem cells derived from fallopian tubes and fallopian tube mucosa according to their biological characteristics and therapeutic potential for treatment of autologous reproductive tract injury.

**Methods:**

Cells isolated from human fallopian tubes and fallopian tube mucosa were expanded and characterised by flow cytometry. The proliferative capacity of both cell types was measured by performing colony-forming unit-fibroblast and Cell Counting Kit-8 assays. Both cell types underwent in vitro adipogenic, chondrogenic, and osteogenic differentiation. The expression of osteocyte-, adipocyte-, and chondrocyte-related genes in the differentiated cell lineages was assessed by reverse transcription-polymerase chain reaction. The secretion of growth factors and immunomodulatory cytokines by both cell types were measured by enzyme-linked immunosorbent assays.

**Results:**

We found that MSCs existed in the fallopian tube mucosa. The comparison between human fallopian tube MSCs (hFTMSCs) and human fallopian tube mucosa MSCs (hFMMSCs) showed that hFTMSCs had a stronger proliferative capacity and shorter duplication time than hFMMSCs. Both cell types could be differentiated into adipocytes, osteoblasts, or chondrocytes in vitro. Real-time polymerase chain reaction analysis demonstrated that hFTMSCs displayed increased expression of osteogenic-specific genes compared with hFMMSCs, but the two types of cells showed no significant increase in the mRNA expression of adipogenic-specific or chondrogenic-specific genes. hFMMSCs and hFTMSCs robustly produced a variety of growth factors and immunomodulatory cytokines.

**Conclusions:**

Human fallopian tube mucosa is a novel source of multipotent cells. hFMMSCs demonstrated stronger proliferative capacity and superior secretion of growth factors and immunomodulatory cytokines than hFTMSCs, making the former a better source of stem cells for the treatment of autologous reproductive tract injury. Compared with fallopian tube, fallopian tube mucosa has more wide-ranging applications and can be used to carry out autologous transplantation.

## Introduction

Mesenchymal stem cells (MSCs) are increasingly found within different post-natal tissues. In 2009, Jazedje et al. showed for the first time that human fallopian tubes are a rich additional source of MSCs and these cells were designated as human tube MSCs (htMSCs) [1]. The studies were of great interest to researchers and clinicians interested in reproduction because they initiated the use of autologous multipotent stem cells derived from human fallopian tubes as a novel source of stem cells for regenerative medicine and they highlighted the usefulness of a material that is typically discarded after surgery. Although human fallopian tubes are a promising source of autologous multipotent stem cells, fallopian tubes must be obtained through a surgical process.

The human fallopian tube is a tubular and seromuscular organ composed of tunica mucosa and two intertwined smooth muscle layers covered by serosa. Fallopian tube mucosa is divided into epithelial lining and the lamina propria [2, 3]. The epithelial lining is uniquely equipped with ciliated and secretory cell types that facilitate ovum pick-up and transport of spermatozoa and ova in opposite directions and that are where fertilization normally takes place. Peg cells are described as stem-like cells and are concentrated on the fimbriated distal end of the fallopian tube [4]. The lamina propria is a layer of loose connective tissue that lies beneath the epithelium and is embedded with a currently unidentified, dispersed network of fibroblast-mesenchymal cells. The fallopian tubes are located between the area where ovulation occurs and the uterus where the zygote is implanted and they act as bridges for sperm and egg transport [5]. The fallopian tube mucosa undergoes periodic changes during the menstrual cycle that result in damage and regeneration [6]. In addition, owing to cyclic ovulatory damage, the fallopian tube must exhibit regenerative activity to rapidly re-establish its normal important reproductive function [7]. The fallopian tube mucosa is similar to endometrium because of its periodic shedding and regeneration during the menstrual cycle throughout a woman’s reproductive life. Fallopian tube mucosa shares the same embryological origin as the endometrium derived from the mucosal lining of the fused mesodermal (paramesonephric) tubes (the Mullerian ducts), which are both dynamic tissues [8]. Previous studies have reported the presence of mesenchymal multipotent cells in many human tissue mucosae, such as endometrium, oral mucosa, intestinal mucosa, ethmoid sinus mucosa, and olfactory mucosa; however, no studies have shown that multipotent stem cells are located in the fallopian tube mucosa [9–14]. Endometrial wound healing involves substantial tissue destruction and subsequent repair and remodelling. Stem cells within the deeper basal layer in the human endometrium that are capable of producing progenitor cells that further differentiate into epithelial, stromal, and endothelial cells as well as growth factors and inflammatory cells play important roles in reconstructing the endometrium [15]. Therefore, we suggested that, similar to the endometrium, multipotent stem cells exist in the fallopian tube mucosa and that fallopian tube mucosa is a novel source of autologous multipotent stem cells. In our opinion, fallopian tube mucosa, which can be obtained by biopsy, is a novel tool that can be used in regenerative medicine. We speculated that multipotent stem cells might exist in the lamina propria of the fallopian tube mucosa. To identify the stem cells within the fallopian tube mucosa and to search for new and alternative sources of MSCs, we identified, isolated, and expanded fallopian tube mucosa multipotent stem cells. Herein, we compared multipotent stem cells derived from fallopian tubes and fallopian tube mucosa according to their biological characteristics and therapeutic potential and assessed their potential for treatment of autologous reproductive tract injury.

## Methods

### Tissue preparation and cell isolation

#### Fallopian tube preparation

Human fallopian tubes (n = 12) were obtained from hysterectomy or tubal ligation/resection samples collected from 35- to 55-year-old fertile women who had not undergone exogenous hormonal treatment for at least 3 months prior to surgery and who had benign disease that did not affect the fallopian tubes. Each patient had undergone bilateral salpingectomy. Informed consent was obtained from each patient, and the experiment was approved by the medical ethics committee of Navy General Hospital.

#### Fallopian tube mucosa preparation

The fallopian tube samples (n = 6) and the mucosa samples (n = 6) derived from the same patients. All of the fallopian tube samples that were acquired from the left side of the patients still contain the mucosa, and the mucosa samples derived from the right-side fallopian tube of the same patient are listed at Table 1. According to a previously published protocol for isolating human amniotic stem cells, human fallopian tube mucosa was obtained from fallopian tubes that would normally have been discarded after tubal ligation/resection [16]. Cell culture media and reagents were purchased from Gibco (Invitrogen, Carlsbad, CA, USA). An entire fallopian tube was placed on a sterile field and longitudinally dissected to expose the fallopian tube mucosa. Then calcium- and magnesium-free Hanks’ balanced salt solution (CMF-HBSS) was added to the mucosa; thereafter, the mucosa was gently massaged to remove blood clots and subsequently peeled using forceps. Each sample was immediately collected in HEPES-buffered Dulbecco’s modified Eagle’s medium/Hams F-12 (DMEM/F-12), maintained at 4 °C, and processed within 24 hours. Informed consent was also obtained from each patient, and the experiment was approved by the medical ethics committee of Navy General Hospital.

**Table 1:** Sources of fallopian tube and mucosa samples

Patient	Age, years	Left side	Right side
1	36	Fallopian tube	Fallopian tube mucosa
2	37	Fallopian tube	Fallopian tube mucosa
3	43	Fallopian tube	Fallopian tube mucosa
4	48	Fallopian tube	Fallopian tube mucosa
5	53	Fallopian tube	Fallopian tube mucosa
6	55	Fallopian tube	Fallopian tube mucosa

#### Cell isolation

All samples were repeatedly washed in CMF-HBSS until all blood cells were removed. The samples were then finely sliced into small pieces, placed inside in a 50-ml Falcon tube, and incubated in 10 ml of trypsin in a reciprocating water bath at 37 °C for 30 minutes. Subsequently, the samples were washed once with 10 ml of DMEM/F-12 supplemented with 10 % fetal bovine serum (FBS) and pelleted by centrifugation at 800×*g*/minute for 8 minutes at room temperature; thereafter, the supernatant was removed with a sterile Pasteur pipette and washed twice with 10 ml of (HBSS) as previously described by Jazedje et al. [1]. After trypsin digestion, further digestion was performed by using 0.1 % collagenase I in a reciprocating 37 °C water bath overnight, and the isolated cells were centrifuged at 400×*g* for 10 minutes and subsequently washed twice with 10 ml of HBSS. The cell pellets were resuspended in DMEM/F-12 (5 ml) supplemented with 10 % FBS, 100 IU/ml penicillin, and 100 IU/ml streptomycin in plastic flasks (25 cm^2^) coated with gluten and maintained in a humidified atmosphere of 5 % CO_2_ in air at 37 °C. The culture medium used for expansion was initially changed every 72 hours and routinely replaced twice a week thereafter. After each passage, the cells were frozen.

### Flow cytometry analysis

For cell surface characterisation, the cells were harvested by using 0.25 % trypsin-EDTA and resuspended in HBSS at a concentration of 1.0×10^6^ cells/ml. The cell suspensions (100 μl) were resuspended in a fluorescence-activated cell sorting tube and stained to evaluate the expression of a variety of markers specific to MSC, hematopoietic, and endothelial lineages; these markers included CD13-FITC, CD29-APC, CD44-PE, CD90-FITC, CD14-APC, CD19-APC, CD34-PE, CD45-FITC, CD73-PE, CD105-PercP CD166-PE, HLA-DR-APC, and HLA-ABC-FITC. Mouse lgG1-FITC, mouse lgG1-APC, mouse lgG1-PE, and mouse lgG1-PercP were used as negative controls. The cells were pelleted by centrifugation at 800×*g*/minute or 5 minutes at room temperature, resuspended in 30 μl of sheath reagent, and mixed with a vortex oscillator. Immunofluorescence antibodies and 100 μl of cell supernatant were added to three Falcon tubes, mixed with a vortex oscillator, and kept in a dark place at room temperature for 20 minutes. One millilitre of sheath reagent was added into each tube, and the cell solution was mixed with a vortex oscillator, centrifuged at 800×*g*/minute for 5 minutes at room temperature, resuspended in 200 μl of sheath solution, maintained at 4 °C in the dark, and tested within 3 hours. All flow cytometry analyses were performed by using Cell Quest software version 3.1 (BD Biosciences, Franklin Lakes, NJ, USA).

### Characteristics of cell proliferation and population doubling time

Cell growth kinetics were measured every day for up to 9 days by using a cell counting kit (Bi-Yun-Tian, Peking, China). The fifth passages of human fallopian tube MSCs (hFTMSCs) and human fallopian tube mucosa MSCs (hFMMSCs) were seeded at 1×10^3^ cells per well in 96-well plates containing DMEM/F-12. Over the following 9 days, 10 μl of Cell Counting Kit-8 (CCK-8) reagent (Beyotime Institute of Biotechnology, Haimen City, China) was added into six wells each day, and the cells were incubated for 4 hours in a CO_2_ incubator until the medium turned yellow. Then cell growth kinetics were measured. The absorbance was determined at 450 and 630 nm by using an auto-microplate reader. With the proliferation curve, the population doubling (PD) time in the logarithmic phase was calculated by using the formula logN/log2, where N is the absorbance of the terminal portion of the logarithmic phase, the cumulative PD level is the sum of the PDs, and the PD rate is the PD divided by time in culture.

### Colony-forming unit-fibroblast assay

hFTMSCs and hFMMSCs were plated at a density of 1,000 cells per well in six-well plates and allowed to grow for 15 days. The cultures were terminated and stained with crystal violet for colony visualization. A colony was defined as a group of cells (>40). The colonies were counted manually under an inverted microscope.

### Differentiation assays

#### Adipogenic differentiation

Passage 5-cultured MSCs were plated at a density of 3×10^4^ cells per well in 24 wells, subjected to adipogenic differentiation induction protocols. Adipogenic differentiation was induced in medium containing DMEM supplemented with 10 % FBS, 100 U/ml penicillin, 100 μg/ml streptomycin, 12 mM L-glutamine, 10 μM insulin, 200 μM indomethacin, 1 μM dexamethasone, and 0.5 mM 3-isobutyl-1-methylxanthine (IBMX) (Sigma-Aldrich, St. Louis, MO, USA). The cells were cultured for 3 weeks, and the medium was changed twice a week. DMEM supplemented with 10 % FBS was used as the control. Accumulation of lipids in these vacuoles was assayed histologically by Oil Red O staining. Briefly, the intracellular accumulation of lipid-rich vacuoles was monitored by staining with 0.3 % Oil Red O solution. First, the cells were fixed in 10 % formalin for 10 minutes; next, they were washed with HBSS and stained with 0.3 % Oil Red O solution for 10 minutes at room temperature. Intracellular lipid-rich vacuoles were stained as red foci.

#### Osteogenic differentiation

MSCs at passage 5 (3×10^4^ cells) were cultured in 24-well plates containing proliferation medium and allowed to attain 70 % to 80 % confluency. In brief, osteogenic differentiation was induced in medium containing low-glucose DMEM, 10 % FBS, 100 μM ascorbic-2-phosphate, 10 mM b-glycerophosphate, and 100 nM dexamethasone (all from Sigma-Aldrich, Gillingham, UK). The cells were maintained in differentiating medium for 28 days, and medium was changed twice a week. At the end of the experiment, the differentiated cells were washed twice with phosphate-buffered saline (PBS) and fixed with 10 % formalin for 10 minutes at room temperature. The cells were then washed thoroughly with PBS and stained with 2 % Alizarin Red S solution (pH = 4 to 4.1), followed by incubation with 0.5 % NH_4_OH for 2 to 5 minutes. The mineralised matrix was identified by the presence of red foci in the stained specimens. The cells were stained with 2 % Alizarin Red S (Sigma-Aldrich, UK) solution to evaluate the mineralised matrix, and the number and size of mineralising nodules were maximised.

#### Chondrogenic differentiation

To induce chondrogenic differentiation, MSCs at passage 5 were plated in 24-well culture plates at a concentration of 1×10^4^ cells/ml and allowed to grow until 70 % to 80 % confluency. The proliferation medium was replaced with chondrogenic medium consisting of high-glucose DMEM supplemented with 0.1 μM dexamethasone, 10 ng/ml transforming growth factor-beta 1 (TGF-β1), 50 μg/ml ascorbic acid and 50 mg/ml insulin-transferrinselenium (ITS) + premix (Becton Dickinson, Franklin Lakes, NJ, USA), 6.25 μg/ml insulin, 6.25 μg/ml transferrin, 6.25 ng/ml selenious acid, and 10 % FBS at 37 °C for 3 weeks. Medium changes were carried out twice a week, and chondrogenesis was assessed at weekly intervals. To determine the presence of glycosaminoglycans within the extracellular matrix, the cells were stained with toluidine blue (Sigma-Aldrich). Briefly, the differentiated cells were fixed with 10 % formalin for 10 minutes at room temperature.

### RNA preparation and quantitative reverse transcription-polymerase chain reaction analysis

Reverse transcription-polymerase chain reaction (RT-PCR) analysis was performed to assess the expression of osteocyte-, adipocyte-, and chondrocyte-related genes in the differentiated cell lineages. Adiponectin, peroxisome proliferator-activated receptor gamma 2 (PPAR-γ2), and adipocyte protein 2 (AP_2_) were used to evaluate the potential for adipogenic differentiation. Osteocalcin, osteopontin, and alkaline phosphatase were related to the osteogenic potential. The chondrogenic potential was assessed on the basis of the expression of type II collagen and aggrecan. Total RNA was extracted by using an RNA Extraction Kit (Bio-Rad Laboratories, Inc., 313 Hercules, CA, USA) in accordance with the instructions of the manufacturer. Before reverse transcription, the extracted RNA samples were treated with RNase-free DNaseI to ensure that the extracted RNA used to synthesize cDNA was free of DNA contamination. RT-PCR analysis was performed to assess the expression of adipocyte-, osteocyte-, and chondrocyte-related genes in the differentiated cell lineages. The extracted RNA samples were subjected to the reverse-transcription reaction by using 1 mg of extracted RNA in accordance with the instructions provided with the RNA-to-cDNA Kit (Bio-Rad Laboratories, Inc., 313 Hercules, CA, USA). The final quantitative RT-PCR contained complementary DNA template, iTaq SYBR Green Supermix with ROX (Bio-Rad Laboratories, Inc., Hercules, CA, USA), and gene-specific primers (Table 2). The following PCR conditions were used: 50 °C for 2 minutes and 95 °C for 10 minutes, followed by 40 cycles of 30 seconds at 95 °C, 45 seconds at 60 °C, and 30 seconds at 72 °C. Beta actin was used as an internal control. The cycle threshold values of beta actin and other specific genes were calculated after the PCR. The normalised fold expression was obtained by using the 2^−△△CT^ method. The results were expressed as the normalised fold expression for each gene.

**Table 2:** Primer sequences for reverse transcription-polymerase chain reaction analysis of differentiation potential

Primers	Forward	Reverse
PPAR-γ2	5′-TTCTCCTATTGACCCAGAAAGC-3′	5′-CTCCACTTTGATTGCACTTTGG-3′
Adiponectin	5′-ATGTCTCCCTTAGGACCAATAAG-3′	5′-TGTTGCTGGGAGCTGTTCTACTG-3′
AP_2_	5′-TGGTTGATTTTCCATCCCAT-3′	5′-GCCAGGAATTTGACGAAGTC-3′
Alkaline phosphatase	5′-ACGTGGCTAAGAATGTCATC-3′	5′-CTGGTAGGCGATGTCCTTA-3′
Osteocalcin	5′-AGAGCGACACCCTAGAC-3′	5′-CATGAGAGCCCTCACA-3′
Osteopontin	5′-CCAAGTAAGTCCAACGAAAG-3′	5′-GGTGATGTCCTCGTCTGTA-3′
Type II collagen	5′-CTGGTATTGCTGGCTTCAAAGG-3′	5′-AGACCATCTTGACCTGGGAAA-3′
Aggrecan	5′-CTGCTTCCGAGGCATTTCAG-3′	5′-CTTGGGTCACGATCCACTCC-3′

### Enzyme-linked immunosorbent assay of growth factors

To evaluate the potency of the growth factor production, each cell type was plated in 75-cm^2^ plastic flasks containing DMEM media without FBS at a density of 1×10^6^/ml for 3 days. The cell supernatants were collected to detect secreted vascular endothelial growth factor (VEGF), epidermal growth factor (EGF), basic fibroblast growth factor (bFGF), hepatocyte growth factor (HGF), and granulocyte-macrophage colony-stimulating factor (GM-CSF) by using human enzyme-linked immunosorbent assay (ELISA) kits (R&D Systems Inc., Minneapolis, MN, USA) in accordance with the instructions of the manufacturer.

### Enzyme-linked immunosorbent assay of immunomodulatory cytokines

To detect the secretion of cytokines related to immunomodulation, the cells were processed as described earlier. The levels of interleukin-2 (IL-2), IL-4, IL-6, IL-10, TNF-α, TNF-β, and interferon-gamma (IFN-γ) were measured by using human ELISA kits (R&D Systems Inc.) in accordance with the instructions of the manufacturer.

### Statistical analysis

Except where otherwise indicated, all of the experiments were repeated three times. Two-sample Student’s *t* test and analysis-of-variance tests were used for statistical analyses, and *P* <0.05 was considered significant. The data were expressed as the mean ± standard deviation of the mean. Statistical analysis of the quantitative RT-PCR data and group comparisons were performed by using the pairwise fixed reallocation randomisation test with the Relative Expression Software Tool (REST 2008). A *P* value of 0.05 was considered statistically significant.

## Results

### Characterisation of cell phenotypes

**Fig. 1:**
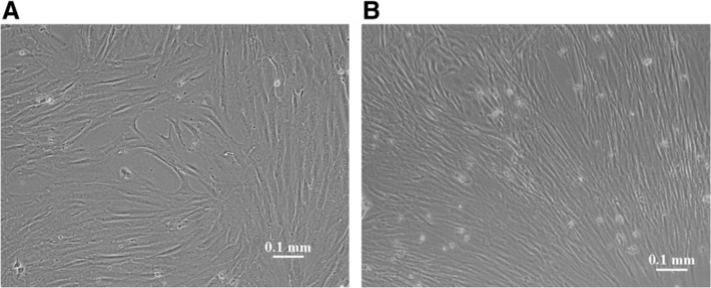
Morphology of human fallopian tube mesenchymal stem cells (MSCs) and human fallopian tube mucosa MSCs. **a** The morphology of the MSCs isolated from human fallopian tube after 12 days of culturing. **b** The morphology of the MSCs obtained from fallopian tube mucosa after 7 days of culturing. Scale bars, 100 μm

**Fig. 2:**
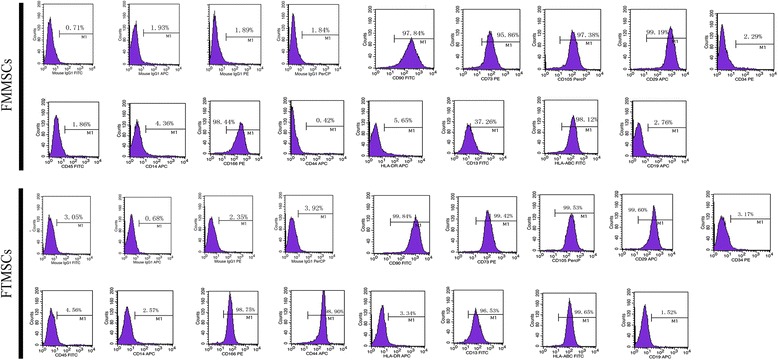
Flow cytometry analysis of cells harvested from human fallopian tube and human fallopian tube mucosa. Mesenchymal stem cells were positive for mesenchymal markers CD90, CD73, and CD105 and cell adhesion molecules CD166 and HLA-ABC and were negative for hematopoietic markers CD34, CD14, CD19, HLA-DR, and CD45. FMMSC, fallopian tube mucosa mesenchymal stem cell; FTMSC, fallopian tube mesenchymal stem cell

Cells isolated from human fallopian tube were morphologically distinguishable from those derived from fallopian tube mucosa. Compared with cells isolated from fallopian tube mucosa, which exhibited spindle-shaped or fibroblast-like morphology, cells isolated from fallopian tubes were larger and had a round, flat shape and nuclear hyperchromatism. The majority of both isolated cell types were adherent to flasks after 24 hours of cultivation. Ten to fourteen and 5 to 7 days were required for the primary cells isolated from fallopian tubes and fallopian tube mucosa to reach confluency, respectively, and the adherent cells formed homogenous cell layers with a whirlpool phenotype (Fig. 1a, b). Flow cytometric analyses were performed to analyse the cells isolated from fallopian tubes and fallopian tube mucosa. The expression profiles of the cells isolated from human fallopian tubes and fallopian tube mucosa and stained with various stem cell markers are presented in Fig. 2. The flow cytometric analyses revealed that hFTMSCs and hFMMSCs were positive for mesenchymal markers (CD90, CD73, and CD105) and cell adhesion molecules (CD166 and HLA-ABC) and were negative for hematopoietic lineage markers (CD34, CD45, CD14, HLA-DR, and CD19). Compared with hFMMSCs, hFTMSCs exhibited higher expression of CD44 and CD13.

### Mesenchymal stem cell expansion and population doubling times

**Fig. 3:**
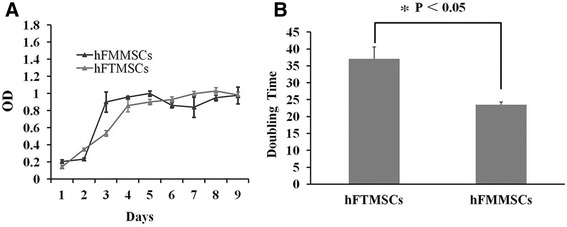
The comparison of proliferative capacity between human fallopian tube mesenchymal stem cells (hFTMSCs) and human fallopian tube mucosa mesenchymal stem cells (hFMMSCs). **a** Comparative analysis of cell proliferative curve between hFTMSCs and hFMMSCs. **b** Comparative analysis of duplication time between hFTMSCs and hFMMSCs. OD, optical density

hFTMSCs began to proliferate immediately after being plated, but the hFMMSCs first underwent a latent phase (the first day). Subsequently, both cell types showed accelerated growth during days 2 to 5 (logarithmic phase) and reached a plateau within 5 to 8 days after culture (Fig. 3a). From the growth curve, we determined that the PD times of fifth-passage hFTMSCs and hFMMSCs in the logarithmic phase were 37.04±3.59 hours and 23.48±0.86 hours, respectively, and a significant difference was found between the values for hFTMSCs and hFMMSCs (*P* <0.05) (Fig. 3b). hFMMSCs have a shorter PD time and greater proliferative capacity than hFTMSCs.

### Plating efficiency

**Fig. 4:**
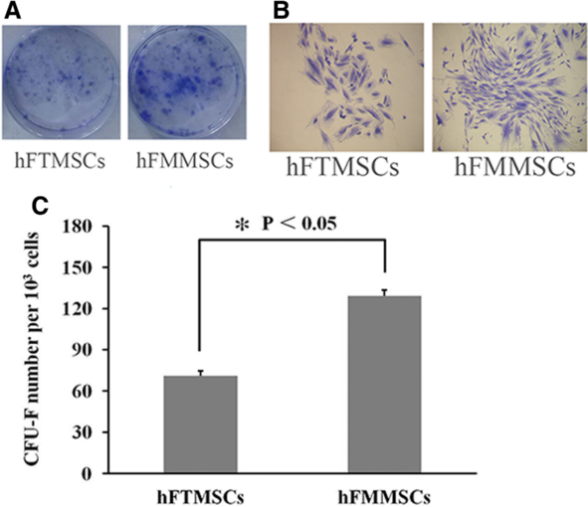
The colony-forming unit-fibroblast (CFU-F) assay of human fallopian tube mesenchymal stem cells (hFTMSCs) and human fallopian tube mucosa mesenchymal stem cells (hFMMSCs). **a** The cells were plated at a density of 1,000 cells per well and cultured for 15 days in six-well plates. **b** The cells were stained with crystal violet dye (0.1 %), and cell colonies with more than 40 cells were counted (×100). **c** The cells were plated at a density of 1,000 per well, and the percentage plating efficiency ([number of colonies counted/number of cells plated] ×100 %) of hFMMSCs was higher compared with hFTMSCs

Cells isolated from fallopian tubes and fallopian tube mucosa were able to grow into colonies. The clonogenic abilities of hFMMSCs and hFTMSCs were determined by colony-forming unit-fibroblast (CFU-F) assays. When these cells were grown at low numbers (1,000 cells/cm^2^) to determine colony-forming units (CFUs), more colonies were formed from hFMMSCs (129.33±4.04) than from hFTMSCs (129.33±4.04) (71±3.61) at passage 5 (Fig. 4).

### Cell differentiation assays

#### Adipogenic differentiation

**Fig. 5:**
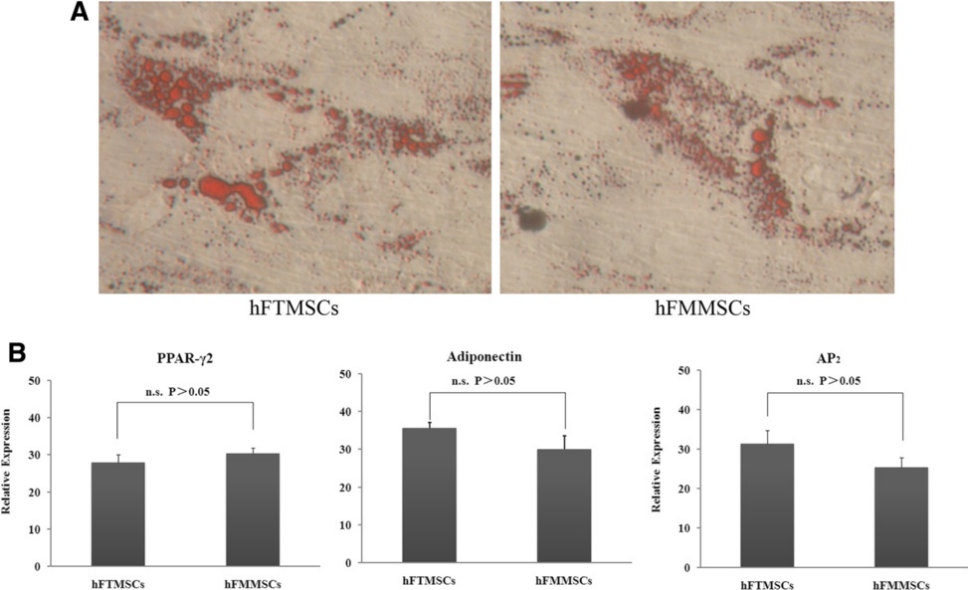
Assessment of adipogenic induction. Both types of mesenchymal stem cells (MSCs) were treated with induction medium for 21 days, and differentiation potential was assessed with quantitative reverse transcription-polymerase chain reaction. **a** Adipogenic differentiation of human fallopian tube MSCs (hFTMSCs) and human fallopian tube mucosa MSCs (hFMMSCs) was stained by Oil Red O (×400). **b** Gene expression profile of induced MSCs. No significant differences were observed in mRNA levels of peroxisome proliferator-activated receptor gamma (PPAR-γ2), adiponectin, and adipocyte protein 2 (AP_2_) between hFTMSCs and hFMMSCs (*P* >0.05)

The potential for adipogenic differentiation was evaluated by using Oil Red O, which stains lipid-rich vacuoles. hFTMSCs and hFMMSCs demonstrated adipogenic potential; however, hFTMSCs displayed similar lipid droplets in the cytoplasm with hFMMSCs (Fig. 5a). The adipogenic potential was quantified by quantitative PCR. Adiponectin, PPAR-γ2, and AP_2_, which are specific adipogenic differentiation factors, were measured to analyse adipogenesis. Compared with hFMMSCs, hFTMSCs had no significant difference in the expression levels of these factors, and this was in accordance with the qualitative results by Oil Red O staining (Fig. 5b).

### Osteogenic differentiation

**Fig. 6:**
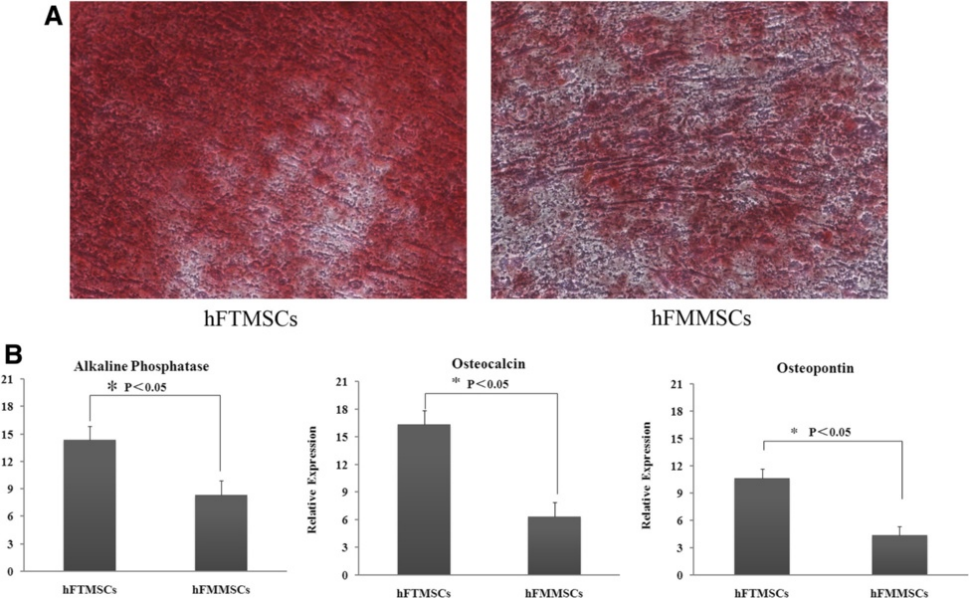
Osteogenic potential of mesenchymal stem cells (MSCs). Both types of MSCs were treated with induction medium for 21 days, and differentiation potential was assessed with quantitative reverse transcription-polymerase chain reaction (RT-PCR). **a** Differences in matrix mineralisation were seen between human fallopian tube MSCs (hFTMSCs) and human fallopian tube mucosa MSCs (hFMMSCs) by staining with 2 % Alizarin Red (×100). **b** Quantitative RT-PCR shows that alkaline phosphatase, osteocalcin, and osteonectin levels were significantly higher in hFTMSCs compared with hFMMSCs (*P* <0.05).

The mineralised matrix was evaluated by 2 % Alizarin Red staining to analyse the osteogenic potential. Both cell types displayed positive staining after 24 days of the pro-osteogenic protocol. No difference was observed between the matrices of two cell types after staining (Fig. 6a). Osteogenic-specific gene expression was measured to quantify the osteogenesis. Compared with hFMMSCs, hFTMSCs displayed a significantly higher expression of the three genes (*P* <0.05) (Fig. 6b).

### Chondrogenic differentiation

**Fig. 7:**
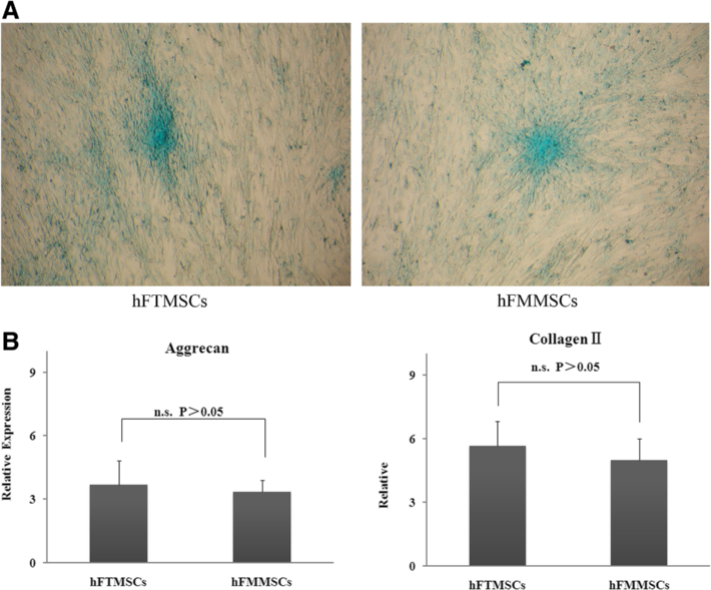
Chondrogenic potential of mesenchymal stem cells (MSCs). Both types of MSCs were treated with induction medium for 21 days, and differentiation potential was assessed with quantitative reverse transcription-polymerase chain reaction (RT-PCR). **a** Abundance of glycosaminoglycans and mucopolysaccharides within the extracellular matrix was demonstrated by the positive Alcian blue staining of induced MSC culture. Both types of MSCs showed chondrogenic induction 21 days after induction (×200). **b** mRNA expressions of aggrecan and collagen type II which were performed by quantitative RT-PCR showed that equivalent mRNA level was observed for aggrecan and collagen between both tissue types (*P* >0.05). hFMMSC, human fallopian tube mucosa mesenchymal stem cell; hFTMSC, human fallopian tube mesenchymal stem cell; n.s., not significant

The chondrogenic potential was estimated by staining with Alcian blue, which demonstrated that both cell types produced extracellular matrix composed of glycosaminoglycans and mucopolysaccharides (Fig. 7a). To quantify the chondrogenesis, we performed RT-PCR, which showed a significant increase in the mRNA expression of aggrecan and collagen type II genes in MSCs treated with chondrogenic differentiation medium compared with untreated MSCs. Additionally, compared with hFMMSCs, hFTMSCs displayed no significant difference in expression of aggrecan and collagen type II (Fig. 7b).

### In vitro secretion of growth factors

**Fig. 8:**
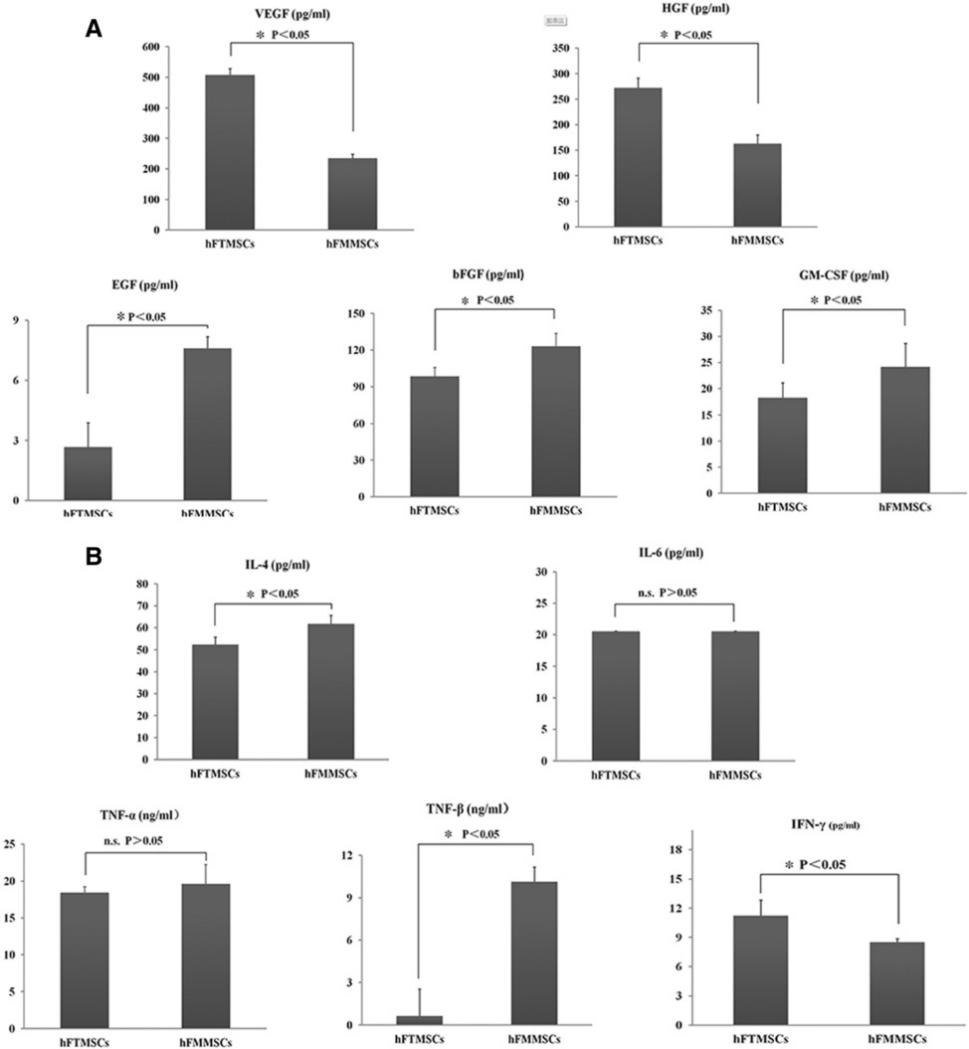
Comparison of in vitro production of growth factors and immunomodulatory cytokines from cultured cells. **a** Concentrations of vascular endothelial growth factor (VEGF), epidermal growth factor (EGF), basic fibroblast growth factor (bFGF), hepatocyte growth factor (HGF), and granulocyte-macrophage colony-stimulating factor (GM-CSF) measured by using enzyme-linked immunoadsorbent assay (n = 3) are depicted. **b** Concentrations of interleukin-4 (IL-4), IL-6, tumor necrosis factor-alpha (TNF-α), TNF-β, and interferon-gamma (IFN-γ) measured by using enzyme-linked immunoadsorbent assay (n = 3) are depicted. hFMMSC, human fallopian tube mucosa mesenchymal stem cell; hFTMSC, human fallopian tube mesenchymal stem cell

Increasing evidence supports the generalisation that cell therapy boosts tissue repair function largely via paracrine mechanisms [26]. The ability of cells isolated from human fallopian tubes and fallopian tube mucosa to secrete large amounts of all growth factors has not been reported. The production of five types of growth factors (VEGF, EGF, bFGF, HGF, and GM-CSF) by hFTMSCs and hFMMSCs was measured and compared. hFMMSCs produced more EGF, bFGF, and GM-CSF than hFTMSCs (*P* <0.05). However, the secretion of HGF and VEGF by hFTMSCs was increased (*P* <0.05) (Fig. 8a).

### Detection of immunomodulatory cytokines

A series of immunomodulatory factors, including cytokines IL-2, IL-4, IL-6, IL-10, TNF-α, TNF-β, and IFN-γ, were measured to estimate the immunomodulatory effects of cultured MSCs on the human immune system and implantation. Cells isolated from human fallopian tube mucosa secreted more IL-4 and TNF-β than cells isolated from fallopian tubes, and hFMMSCs produced lower amounts of IFN-γ than hFTMSCs (*P* <0.05). No significant difference in the secretion of IL-6 and TNF-α was found between hFTMSCs and hFMMSCs. Owing to their low concentrations, IL-2 and IL-10 could not be detected (Fig. 8b).

## Discussion

Bone marrow-derived MSCs (BM-MSCs) are still recognized as stem cell banks in the area of regenerative medicine. However, more and more tissues are found to host MSCs, and a search for alternative sources of MSCs to replace BM-MSCs is an important aspect to consider for regenerative medicine applications [17–21]. Fallopian tubes, which are normally discarded in surgical procedures, are a source of MSCs for regenerative medicine and can be used to carry out autologous transplantation, but there are certain limitations: (1) The supplication is limited because only a few patients have the request for tubal resection, and (2) if fallopian tube is used for autologous transplantation, its scope of application will be limited. Not everyone wants to remove their own fallopian tube tissue if unnecessary.

In 2009, Jazedje et al. showed for the first time that human fallopian tube is a rich additional source of MSCs and these cells were designated as htMSCs [1]. In 2012, Jazedje et al. reinforced the idea that the autologous hFTMSCs transplanted into non-immunosuppressed rats can successfully enhance bone regeneration in vivo and have potential use for treatment of osteoporosis and bone reconstruction or regeneration [22]. In 2013, Indumathi et al. confirmed again the existence of hFTMSCs. They also made a comparison of hFTMSCs and BM-MSCs in terms of their biological characteristics and found that hFTMSCs could replace the BM-MSCs and be additional stem cell sources for regenerative medicine [8]. We found that, in the previous study, the human fallopian tubes were obtained from hysterectomy or tubal ligation/resection and that the whole layers of fallopian tube are digested to isolate the MSCs. The fallopian tube can be divided into three layers from inside out: tunica mucosa and two intertwined smooth muscle layers covered by serosa. Although our study focused on an innovative method, we try to strip the fallopian tube mucosa absolutely out of the whole fallopian tube tissue and then have a digestion of the stripped fallopian tube mucosa to isolate the MSCs, and we suggest that this cell source is suitable for regenerative medicine and has a broader application than hFTMSCs. Fallopian tube mucosa can be obtained by the salpingo-scope, which is a less invasive procedure than tubal resection [23]. The vaginal route is the least invasive and most economical route and should be the first choice to obtain the hFMMSCs. Although our study did not get the fallopian tube mucosa through the salpingo-scope, we will take it into consideration in our further study.

In this study, we performed a head-to-head comparison of the two kinds of stem cell types for autologous transplantation by assessing multiple in vitro parameters. hFMMSCs are smaller and thinner than hFTMSCs and can be mixed with other unidentified cells derived from the seromuscular layer. Thus, our novel isolation of purified multipotent cells from fallopian tube mucosa is meaningful. Our study demonstrated that hFMMSCs have a more powerful proliferative capacity than hFTMSCs. hFTMSCs displayed superiority over hFMMSCs in bone formation, but the two types of cells showed no significant increase in the mRNA expression of chondrogenic- and adipogenic-specific genes. hFMMSCs and hFTMSCs robustly produced a variety of growth factors, including bFGF, EGF, GM-CSF, HGF, and VEGF. Importantly, hFMMSCs secreted more bFGF, EGF, and GM-CSF than hFTMSCs, all of which are involved in the re-epithelialisation.

To the best of our knowledge, this is the first report on the isolation of cells from fallopian tube mucosa, and these cells adhered to the minimum criteria proposed by the Mesenchymal and Tissue Stem Cell Committee of the International Society for Cellular Therapy for defining human MSCs. These criteria consist of the following three points: (1) MSCs must be plastic-adherent; (2) they must express CD105, CD73, and CD90 and lack expression of CD45, CD34, CD14 or CD11b, CD79a, CD19, and HLA-DR surface molecules; and (3) they must be able to differentiate into osteoblasts, adipocytes, and chondroblasts in vitro [24]. Our study found that hFMMSCs have a more powerful proliferative capacity than hFTMSCs and demonstrated its great application value. The required cell number for cell therapy is 1×10^6^/kg; for an adult weighing 50 kg, the total cell number required for therapy is 5×10^7^. So achieving a sufficient amount of cells in a short period of time is important in order to achieve the best treatment time, especially for autologous transplantation. MSCs with a high proliferative ability can shorten the in vitro culture time. Thus, we can achieve a standard amount of cells in an extraordinarily short time.

hFMMSCs can be a supplement of autologous transplantation source and broaden the application scope, especially for the treatment of autologous reproductive tract injury. Fallopian tube mucosa may the best source of material for endometrial reconstruction and restoration of reproductive function because of its ability to mediate blastocyst adhesion to the fallopian epithelium leading to pregnancy [25]. Furthermore, fallopian tube mucosa may be a good source of material because of its similarity to the surrounding tissue. The fallopian tube and uterus share the same embryological origin and both are dynamic tissues [6, 8].

There is evidence that the mechanisms involved in the therapeutic properties of tubal mucosa include not only the ability of the tubal mucosa multipotent stem cells to differentiate into endometrium to replace the injured cells but also their secretion of factors that promote endometrium repair in a paracrine manner. These factors are divided into six categories: immunomodulation, anti-apoptosis, angiogenesis, support of the growth and differentiation of local stem and progenitor cells, anti-scarring, and chemoattraction to promote tissue repair [26]. MSCs also attenuate the pathological remodeling, including scar formation, of many tissues, such as the heart, airways, and skin [27]. Our study showed that hFMMSCs and hFTMSCs robustly produced a variety of growth factors, including bFGF, EGF, GM-CSF, HGF, and VEGF. Importantly, hFMMSCs secreted more bFGF, EGF, and GM-CSF than hFTMSCs. EGF and bFGF were reported to have significant promoting effects on the migration and proliferation of functional cells in wound healing. EGF has been found to play a critical role in the wound healing process [28–30]. Based on this, we observed that hFMMSCs demonstrated their superiority to hFTMSCs in re-epithelialisation. The secretion of immunomodulatory factors, including the cytokines IL-4, IL-6, TNF-α, TNF-β, and IFN-γ, by both cell types demonstrates the immunomodulatory function of the cells. Compared with hFTMSCs, hFMMSCs secrete more of the anti-inflammatory cytokine IL-4 and less of the pro-inflammatory cytokine IFN-γ, and this indicates that hFMMSCs have a stronger anti-inflammatory role, making them particularly beneficial in chronic wound treatment because they advance the wound past a chronic inflammatory state into the next stage of healing. Therefore, hFMMSCs are a better stem cell source than hFTMSCs for the treatment of autologous reproductive tract injury mainly because of their rapid cell expansion, which is the decisive factor for the assessment of the therapeutic potential of autologous therapies. hFMMSCs can be produced in ample numbers soon after injury. The cells should be available and should be acquired in ample numbers immediately after injury. In addition, their powerful secretion of growth factors, which is crucial for re-epithelialisation, is beneficial.

These results inspired a search for autologous multipotent stem cells derived from human fallopian tube mucosa. In summary, the mucosa of all tissues will be the best source of multipotent stem cells because the multipotent stem cells in the complete tissue mucosa are superior to those of the whole tissue. Mucosa, which forms the lining of many body cavities such as the digestive, respiratory, and reproductive tracts, acts as a barrier to the outside environment and has powerful regenerative ability. Tissue mucosa can be uneventfully harvested from patients in a minimally invasive, virtually painless manner with low morbidity. All human tissue mucosa can be obtained by biopsy rather than through surgical procedures. Thus, we suggest that MSCs isolated from tissue mucosa are a novel, promising, and abundant source of MSCs that shows superior potential for cell therapy because of the straightforward, easy, and minimally invasive collection; high proliferative capacity; and immunomodulation and tropism for the healing of injuries. Furthermore, autologous transplantation can overcome the traditional hazards associated with allogenic transplantation. So we proposed that MSCs isolated autologous mucosa may be best source of MSCs. Therefore, we propose also that autologous mucosa, which has the regenerative capacity to recover its integrity after biopsy, may be the best source of MSCs.

All in all, we focus especially on the comparison of stem cells acquired from two different methods. Fallopian tube mucosa can be acquired by salpingo-scope, and we have demonstrated the existence of MSCs in our study. Although in our experiments we did not use the tool of salpingo-scope to get fallopian tube mucosa, we at least show that fallopian tube mucosa is a better stem cell source than the whole fallopian tube in many ways. So we proposed to obtain fallopian tube mucosa by salpingo-scope to replace tubal resection. We also successfully isolated MSCs from the fallopian tube, but tubal resection is more invasive than salpingo-scope and is associated with more discomfort for the patient. In addition, we found that hFTMSCs are inferior to hFMMSCs according to their biological characteristics and therapeutic potential. We concluded that regardless of the acquisition method and their biological characteristics and therapeutic potential, the fallopian tube mucosa is a better MSC source than the fallopian tube.

## Conclusions

In our study, we make a comparison of hFTMSCs with hFTMSCs to find a better stem cell source to repair human reproductive tract. Although these data showed that hFTMSCs demonstrated stronger proliferative capacity and superior secretion of growth factors and immunomodulatory cytokines and we also found that multipotent stem cells derived from tissue mucosa have advantages over the whole tissue because of their minimally invasive collection method, high proliferative capacity, immunomodulation and tropism for the healing of injuries as well as autologous transplantation can also be realised by using tissue mucosa. Fallopian tube mucosa is a convenient and readily available source of stem cells, and the harvest procedure is less invasive than tubal resection and is associated with little discomfort for the patient. Fallopian tube mucosa has wide-ranging applications and can be used to carry out autologous transplantation. hFMMSCs demonstrated an obvious superiority in clinical applications, and especially for the repair of autologous reproductive tract injury, fallopian tube mucosa performed the potential application.

## Abbreviations

AP_2_: Adipocyte protein 2
bFGF: Basic fibroblast growth factor
BM-MSC: Bone marrow-derived mesenchymal stem cell
CMF-HBSS: Calcium- and magnesium-free Hanks’ balanced salt solution
DMEM/F-12: Dulbecco’s modified Eagle’s medium/Hams F-12
EGF: Epidermal growth factor
ELISA: Enzyme-linked immunosorbent assay
FBS: Fetal bovine serum
GM-CSF: Granulocyte-macrophage colony-stimulating factor
hFMMSC: Human fallopian tube mucosa mesenchymal stem cell
hFTMSC: Human fallopian tube mesenchymal stem cell
HGF: Hepatocyte growth factor
htMSC: Human tube mesenchymal stem cell
IFN-γ: Interferon-gamma
IL: Interleukin
MSC: Mesenchymal stem cell
PBS: Phosphate-buffered saline
PCR: Polymerase chain reaction
PD: Population doubling
PPAR-γ2: Peroxisome proliferator-activated receptor gamma 2
RT-PCR: Reverse transcription-polymerase chain reaction
TNF: Tumor necrosis factor
VEGF: Vascular endothelial growth factor
